# Skimmed Milk Applied as a Phytopharmaceutical Product: A Risk for Allergic Populations?

**DOI:** 10.3390/ijerph18052400

**Published:** 2021-03-01

**Authors:** Halshka Graczyk, David Vernez, Nenad Savic, Antoine Milon, Eric Masserey

**Affiliations:** 1 Center for Public Health and Primary Care Medicine (Unisanté), University of Lausanne, 1011 Lausanne, Switzerland; graczyk@ilo.org (H.G.); Nenad.Savic@unisante.ch (N.S.); Antoine.Milon@hopitalvs.ch (A.M.); 2 Public Health Service, Vaud Canton, 1014 Lausanne, Switzerland; eric.masserey@vd.ch

**Keywords:** risk assessment, plant protection products, lactose, milk protein, milk allergy, treatment, air measurements, surface contamination, modelling

## Abstract

Milk allergy is among the most common food-related allergies. Milk-based products are recognized as plant protection products (PPPs) in several countries as alternatives to synthetic pesticides. The potential health risk for allergic workers, as well as the general population, is yet to be assessed. An investigation was conducted in the Vaud Canton of Switzerland, where milk-based products are sprayed by helicopter over vineyards. Air lactose concentration was measured at 14 locations via 25 mm IOM Multidust samplers. Residual lactose concentration was measured on the surface of leaves over 7 days following spraying. Surface contamination downwind from the treated area was estimated through computer-based modeling using AgDRIFT^®^ software. The average milk protein concentration inside and outside the vineyard was 0.47 and 0.16 µg/m^3^, respectively. Milk residues persisted on the leaf surface for an average of three days. Modelling results revealed an estimated order of magnitude of 0.1–0.5 µg/m^3^ in milk proteins within one hour after the treatment in the close vicinity of the treated area. Our results reveal that the potential exposure to milk proteins in and around helicopter-treated vineyards is not negligible and that prevention messages targeted to individuals with severe allergies should be considered.

## 1. Introduction

### 1.1. Milk Allergy

Food allergies have become a concrete and widespread public health problem, reaching “near-epidemic proportions” in some regions of the world [1]. Cow milk allergy is among the most common food allergies, affecting between 0.6% to 3% of children below the age of 6 years [2]. Approximately 2.6% of infants and young children in North America have been reported to suffer from milk allergy [3,4]. In most children, approximately 80%, outgrow their milk allergy by the age 16 years; however, approximately 15% of affected children remain allergic throughout adulthood [2,5]. In Switzerland, allergy to cow’s milk is the second most frequent food allergen in infancy, the first being eggs [3]. IgE mediated reactions can lead to urticaria, angioedema and gastrointestinal symptoms. The severe end of the spectrum can include life-threatening anaphylaxis with airway, breathing or circulatory involvement. Non-IgE mediated reactions include cutaneous and gastrointestinal symptoms that could be persistent, severe or treatment resistant [6].

Cow milk is the third most common cause of foodborne anaphylaxis in children and adults, after peanuts and nuts. Milk is also the third most common cause of fatal or almost fatal anaphylactic reactions (8% to 15% of cases) [7]. Acute allergic responses in patients with milk allergy are due to IgE directed responses against different milk allergens. All proteins from cow milk are potentially allergenic and several milk protein polysensitivity occurs in most patients [2].

Food allergies, including those to milk proteins, often occur after consuming the allergen. However, individuals who are allergic to food proteins by ingestion may also react to the same proteins by inhalation [8]. Hypersensitivity reactions after inhalation of food particles as a primary cause for food allergy is an increasingly recognized problem in children [9]. The inhaled quantity can be substantial, particularly in certain occupational settings, or very trivial, such as mere smelling. While inhalation is commonly recognized as symptom-provoking in individuals who have previously developed food allergy, inhalation can also cause de novo sensitization [8], especially in occupational settings. A cross sectional study from Thailand showed that workers (N = 167) occupationally exposed to milk powder by inhalation were at increased risk of nasal symptoms, wheezing and breathlessness, and exhibited reduced spirometric lung function, even at relatively low air concentrations of milk dust [10].

Asthma is a strong predisposing factor for severe reactions to cow milk allergy. In the majority of patients, food particle inhalation induces respiratory symptoms that can be nasal (rhinorrhea, sneezing and nasal congestion), ocular (tearing, redness and irritation) or lower respiratory (coughing and wheezing). In addition, skin manifestations and even, although much more rarely, anaphylaxis can occur [8]. It is worth noting that a proportion of particles inhaled through the mouth may impact the back of the throat, and may subsequently be swallowed [11].

### 1.2. Milk Used as a Plant Protection Product

Milk-based products are sometimes used as Plant Protection Products (PPPs) alone or in addition to PPPs, to act as fungicides, due to their properties to combat mildew. Milk-based products are also known for their use as wetting or adhesive agents, thus allowing other substances to adhere to plant surfaces. Although this alternative is not yet widespread, milk-based products to replace synthetic pesticides, specifically for the treatment of sensitive and lucrative crops, such as grapes used for winemaking. In Switzerland, out of a total of 8200 hectares treated by helicopter in 2020, approximately 1800 hectares were treated with a solution containing skimmed milk (source: Federal Office for the Environment). The milk dosage used for these treatments was 10 kg/ha.

In Switzerland, PPPs are regulated by the Ordinance on Plant Protection Products (OPPh in French; Ordonnance sur la mise en circulation des produits phytosanitaires) [12], to ensure that they are used in accordance with the requirements, and that they do not have unacceptable health effects for humans, animals or on the environment. Skimmed milk (or lean milk) and whey are considered to be basic substances and are thus listed in Annex 1 of the OPPh. These products are often purchased at the local grocery store, and represent the exposure of interest for this case study (French: lait maigre; lait drink).

Milk proteins account for approximately 3.2%–3.8% of milk. They consist of about 20% whey proteins with major components α-lactalbumin (α-LA), β-lactoglobulin (β-LG) and 80% caseins, divided into major subclasses α-(αS1- and αS2-), β-, and κ-casein (-CN), which are arranged in micelles [13].

While the use of natural products in lieu of chemical pesticides can be seen as a positive trend, the potential health risks for allergic workers, as well as the general population, has yet to be investigated. As such, the aim of this research was to implement the first known risk assessment of milk applied as a PPP for allergic populations.

## 2. Materials and Methods

An investigation, combining field measurements and modelling, was used to assess the potential risk for allergic populations exposed to milk applied as a PPP. In regard to hazard identification, the hazard or exposure of interest is bovine proteins contained in skimmed milk (maximum 0.5% fat content) applied to vineyards by helicopter dispersion.

### 2.1. Field Measurements

**Figure 1:**
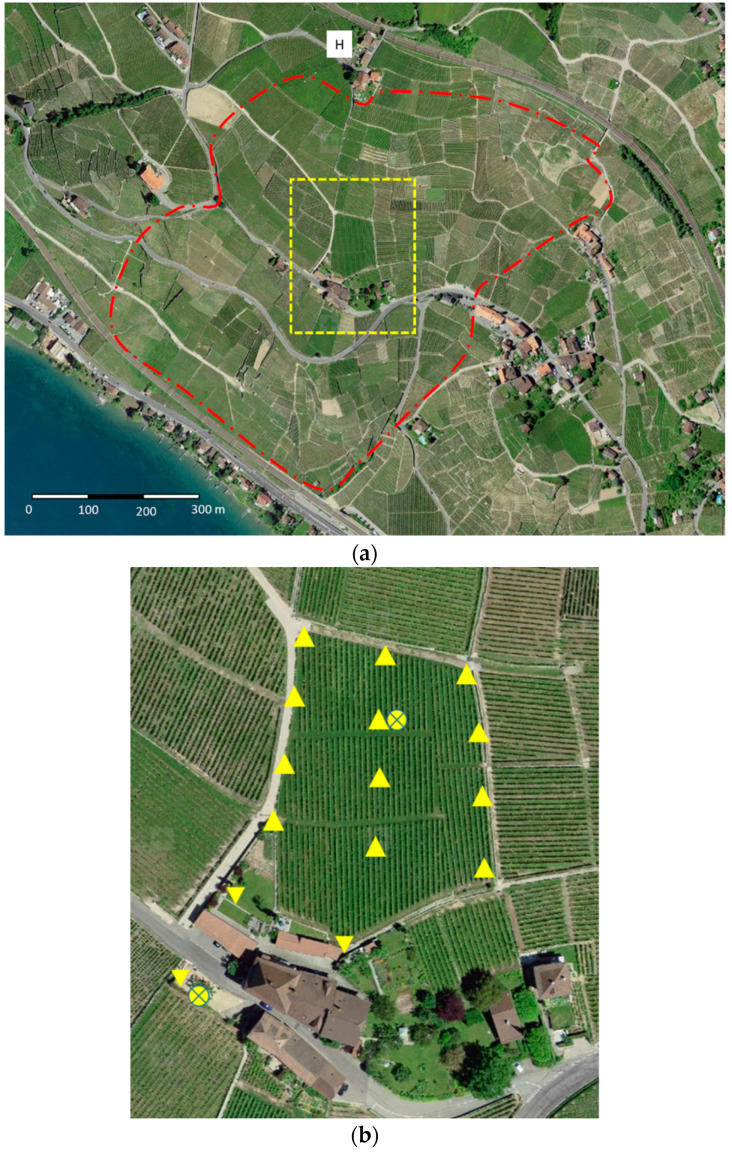
Area treated with skimmed cow milk on 7 June 2018: (**a**) overview of the treated area; (**b**) measurement area: △ sampling location inside of the treated area, ▽ sampling location outside of the treated area, ⊗ direct-reading air measurement location.

The field measurements were carried out on 7 June 2018, between 11:00 a.m. and 1:00 p.m., during the skimmed milk treatment of a vineyard located in the Lavaux region (canton of Vaud, Switzerland, 46°29′57.8″ N 6°42′23.5″ E). The treated area is shown in Figure 1a. This region is very steep, which makes access to vehicles in the vineyards difficult and makes treatment by helicopter attractive despite its cost. Treatment of the area took about 2 h, during which time the helicopter treated about 50 hectares, flying back and forth between the treated vineyards and the refill area (marked H on the map). The helicopter used on the day of the treatment was a AS350 B2 (Airbus Helicopters, France). 

Field measurements were carried out in a vineyard of about 4000 m^2^, located in the middle of the treated area, at a volunteer winegrower’s property (Figure 1b). Quantitative field-based sampling of air was collected at 15 locations (12 inside the treated perimeter, 3 outside). Airborne concentration was measured inside the vineyard perimeter to provide proxy estimates for worker exposure. Measurements outside the vineyard were taken to represent the exposure of the general population under unfavorable conditions (living area adjacent to the vineyard, public road). Outside measurements were taken. Residual milk was also measured on the surface of leaves over 7 days following spraying in order to quantify temporal degradation. A more detailed view of the sampling area, numbering the sampling locations, is provided in the Supplementary Material (Figure S1).

### 2.2. Airborne Concentrations

Aerosol samples were taken during the treatment of the selected vineyard. The pumps were laid out in the field as described in Figure 1b. The three measurement points outside the treatment zone were intended to assess the dissemination of the products by the wind, the helicopter not having the right to spray close to the houses ensuring indirect contact of these samples with the treatment product. The sampling started immediately prior to the treatment and for a period of about 160 min.

Air samples were taken using SKC pumps (224-PCXR4, SKC Inc., Eighty Four, PA, USA) set at a flow rate of 2L/min and equipped with 25 mm IOM Multidust samplers (SKC Inc., PA, USA) adapted to measure the inhalable fraction of aerosols in the air. A fiberglass filter 25 mm 1.0 micron (N° 225–702, SKC Inc., PA, USA) in diameter was used as a sampling medium to collect non-volatile milk material. 

In the laboratory, the fiberglass filters were extracted with 2 mL of milliQ water. The vials were put in a bath at 60 °C for 30 min and stirred for 10 min with a rotary shaker. The solutions were filtrated and injected in a ICS-5000 ion chromatography system (Thermo-Dionex, Grand Island, NY, USA) equipped with a DP 5000 pump, an autosampler AS-AP and a thermostatized compartment DC 500 with an electrochemical detector. The analytical column was a carbopac PA1 (50 + 250 mm × 2.0 mm, Dionex Grand Island, NY, USA), maintained at a temperature of 30 °C. The mobile phase was 150 mM NaOH at a flow rate of 0.4 mL/min in isocratic mode. The system was calibrated using a stock solution of 1.5 g Lactose/L, obtained in dissolving lactose standard from Sigma Aldrich (Buchs, Switzerland) in water milliQ. Lactose was used as a marker of milk concentration in the air and on the leaves. This marker was chosen because of the sensitivity and reliability of its determination in ion chromatography. The lactose concentrations obtained were then converted to milk protein concentration according to Equation (1).

(1) $$Milk\;protein\;conc.=\;\frac{protein\;\%\;in\;skimmed\;milk\;}{lactose\;\%\;in\;skimmed\;milk\;}\cdot Lactose\;conc.\;measured$$

Direct-reading air concentrations were also conducted in two locations during the treatment period. Two nephelometers (pDR-1000, Thermo Electron Corp., Beverly, MA, USA) were placed in the treated area and outside the treated area, in parallel with filter samples (Figure 1b). These devices record the aerosol concentration in the air, continuously, as a function of time. Optical measurements from nephelometers are not specific to a particular substance and must, therefore, be adjusted to the cow milk protein concentration using the results of the filter samples obtained at the same location. They are, therefore, essentially semi-quantitative measures, which are not subject to statistical analysis. They allow visualizing a profile of concentration during the treatment, in order to appreciate the persistence of the aerosols during the treatment and during the hours following the passage of the helicopter.

### 2.3. Surface Contamination

Grape leaves were harvested immediately after treatment and at successive intervals on the days following treatment (D, D + 1, D + 3 and D + 7). A sample at D + 10 was planned, but a new treatment was given that day. The same sampling locations as for air measurements were used for leaves sampling (Figure 1b). The leaves were chosen randomly, without paying attention to the presence of droplets or white spots. The criterion was to choose leaves from the upper part of the plant and medium in size. Several people participated in the harvest of the leaves introducing a possible effect of involuntary selection.

The collected leaves were put in plastic bags, identified by date and location and kept in the fridge. Residues on the grape leaves were extracted directly in the collection bags with 40 milliliters of milliQ water. The bags were put 30 min in a 60 °C bath prior to filtration and injection, using the same analytical method as that used for filters. After scanning the leaves for surface determination, the amount of lactose measured on the leaves was converted to an amount of cow milk proteins per unit area to determine the persistence of milk residue as a function of time on the surface of the leaves and its geographical spread during treatment.

Surface contamination far from the treated area (downwind concentrations) was estimated through computer-based modeling using AgDRIFT^®^ software (version 2.1.1, US EPA, Washington, NW, USA). AgDRIFT^®^ is a drift model used to assess the movement and deposition of pesticides sprays. It is primarily used as a decision-making tool for low-flight applications. The model requires a list of physical input parameters that are used to calculate evaporation of the liquid phase from particles and to account for wind speed, meteorological conditions, the spraying rate and the helicopter/airplane type and characteristics. The parameters used in this study are given in Table 1. Realistic conservative estimates were used for most parameters, but ranges were considered for two parameters (boom height and wind speed) which may have varied significantly during treatment.

## 3. Results

Considering that all milk proteins are potentially allergenic, all results are expressed here in mass concentrations of milk proteins, assuming that the total protein and lactose content in skimmed milk is of 3.4% and 5.1% in mass, respectively [14].

### 3.1. Airborne Concentrations

**Figure 2:**
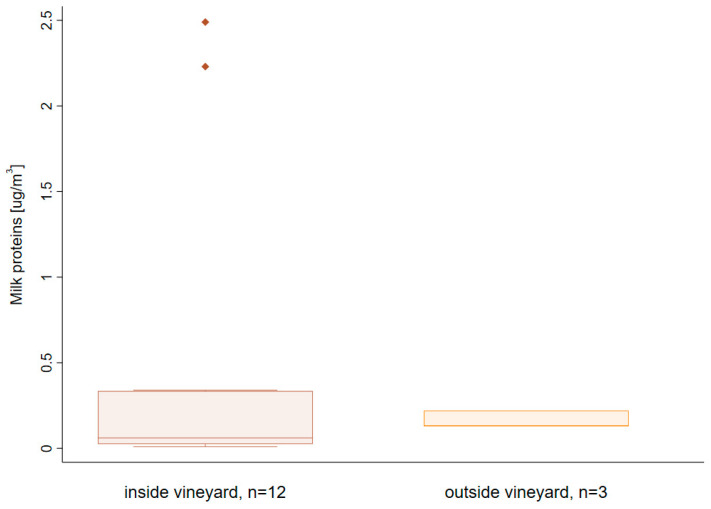
Cow milk proteins concentrations in air during treatment (t = 160 min) inside and outside the vineyard.

Air concentrations measured during and shortly after treatment are shown in Figure 2. The average concentration in cow milk proteins observed over all measurements was 0.41 μg/m^3^ (n = 15, SD 0.8 μg/m^3^), with average concentrations inside and outside the vineyard of 0.47 and 0.16 μg/m^3^, respectively. This difference is mainly due to the greater variability of concentrations inside the vineyard, illustrated by the presence of two outliers in the order of magnitude of 2 μg/m^3^. Apart from these two outliers, it is interesting to note that the air concentrations in the living area near the vineyard (0.13–0.22 μg/m^3^) were close to those observed in the field.

**Figure 3:**
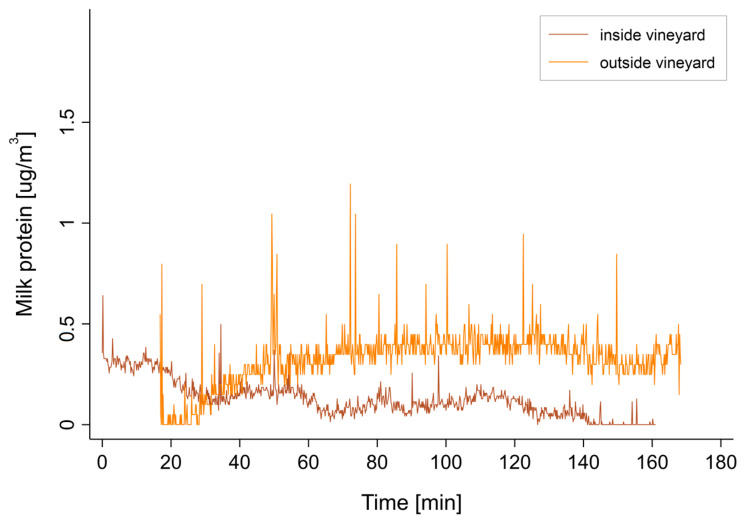
Milk proteins concentration in the air, measured by nephelometer inside and outside of the treated area.

The temporal evolution of air concentrations during and immediately after treatment was measured at two locations, representing the inside and outside of the treatment perimeter, respectively (Figure 3). Air concentrations of 0.5 μg/m^3^ were observed inside the vineyard at the beginning of the treatment. However, these decreased rapidly under the combined effect of sedimentation and wind entrainment of aerosols. Conversely, aerosol concentrations outside the vineyard could only be measured several minutes after the start of treatment. After about 45 min, a concentration similar to that observed during treatment inside the vineyard was reached. It is interesting to note that the concentration outside the field did not decrease during the next two hours of measurements. This effect is probably due to the fact that it is the finest aerosols, and thus those with the lowest sedimentation rate, that are carried by the wind. This also means that concentrations of the same order of magnitude as those observed in the field can persist for several hours outside the treatment area.

Note that the nephelometric measurement represents only the inhalable fraction of the aerosol and, therefore, probably underestimates the total quantity of milk proteins deposited in the vineyard area. The large droplets tended to impact the soil or leaves directly without being carried by the wind or being able to enter the respiratory tract.

### 3.2. Surface Contamination

**Figure 4:**
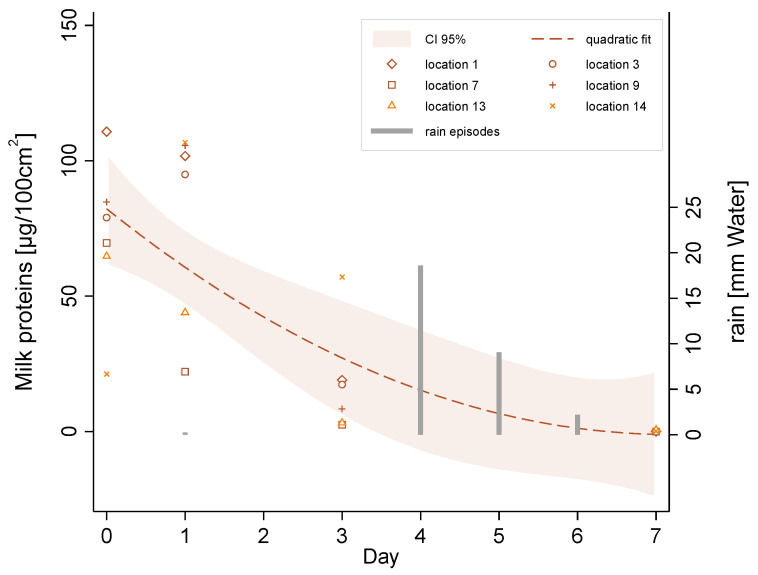
Milk proteins surface concentration on leaves and rain episodes as a function of time inside the vineyard.

Results of the surface sampling on the leaves are shown in Figure 4. A large variability was observed between the different leaves, suggesting an inhomogeneous spread of the skimmed milk. A visual examination of the treated leaves showed that the milk deposits were in the form of irregular spots (see picture of treated leaves in supplementary material). The upper leaves are generally treated more heavily than the lower leaves. The treatment of leaves at the same height can also be uneven, as it depends on their respective position and orientation at the time of application. This explains why the concentrations measured on the same vine plant can sometimes increase over the time between two close samples. This effect was, for example, visible for location 3 between day 0 and day 1.

Overall, the milk deposits showed some persistence during the first 3 days, with concentrations of the order of 10–100 μg/100 cm^2^, and low persistence in the medium term, with concentrations not exceeding 2 μg/100 cm^2^ after 7 days. It should be noted that rainfall events during days 4, 5 and 6 probably contributed to the accelerated removal of the milk deposits from the leaves.

**Figure 5:**
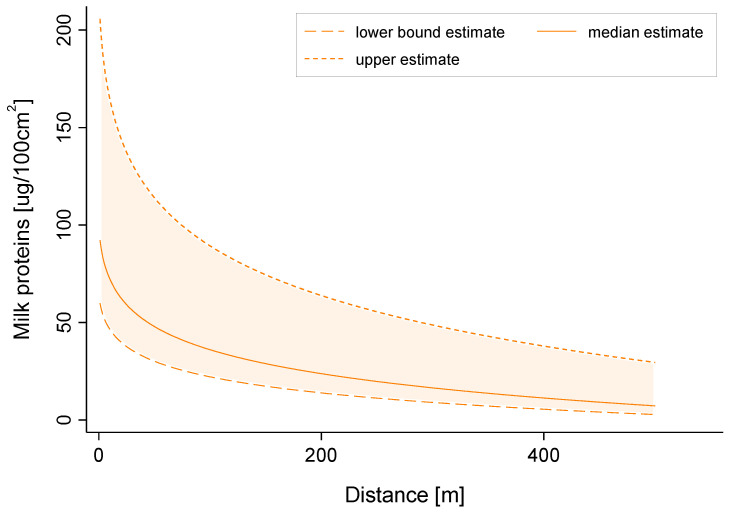
Simulated milk proteins surface concentration at ground level as a function of downwind distance from the vineyard.

As shown in Figure 5, the simulation of surface deposition conducted with AgDRIFT showed initial field edge concentrations very close to those measured in the vineyard. The median value of the simulation of about 100 μg/cm^2^ was similar to the upper bound of the confidence interval of the measured values. The decrease in surface contamination was exponential, with a sharp drop in the first 100 m and a residual surface concentration at 500 m of less than 10 μg/cm^2^. In the most conservative scenario (upper estimate), the concentrations were significantly higher than in the median scenario, ranging from 2 to 4 times the concentrations observed in the median scenario at 0 and 500 m, respectively.

## 4. Discussion

This study assessed whether milk applied as a PPP is a risk for allergic populations, namely, children who are most likely to suffer from the most adverse effects. This question is particularly relevant in regions where vineyards are located in tourist areas or close to inhabited areas, where it is difficult to prevent the presence of the public near the treatment area.

Aerosol sampling revealed an estimated average concentration of 0.47 and 0.16 µg/m^3^ in milk protein inside and outside (but close) the vineyard, respectively. It should be noted that the difference between the two zones is essentially due to the high variability observed in the inner zone of the vineyard. These results are consistent with direct-reading measurements, which showed an increasing aerosol concentration near the treated area, with a maximum of about 0.4 µg/m^3^ about 45 min after the start of treatment. The air concentration at a distance from the vineyard is difficult to estimate due to the complex geography of the area (steep slope). Simulations conducted with AGDrift, which essentially calculates surface deposition, however, suggested an exponential decrease, with a strong abatement during the first 100–200 m. It is, therefore, reasonable to estimate that the order of magnitude of milk protein concentration, during and within one hour after treatment on public areas in the close vicinity of the treated area (roads, paths and outside dwellings), is within an (conservative) order of magnitude of 0.1–0.5 µg/m^3^ (or ng/L-air).

The threshold dose of inhaled milk protein was not established. However, it is well known that the inhalation of cow’s milk can induce severe anaphylactic reactions in allergic individuals [15]. Uncertainty in the dose response makes it difficult to fully characterize the potential health impacts for exposure levels presented in the study. Nevertheless, evidence from the literature has shown that repeated exposure to low doses of inhaled milk proteins might also exacerbate chronic airway inflammation and lead to poor asthma control in the absence of acute immediate reactions [16]. Considering the high vascularity of the respiratory tract mucous membranes and lack of protective mechanisms unique to the gastrointestinal tract, such as digestive enzymes and low pH, one could hypothesize that smaller doses of inhaled food allergen would induce an allergic reaction compared with an ingested allergen in highly sensitized/allergic individuals [16]. It is also important to consider that while inhalation is commonly recognized as symptom provoking in individuals who have previously developed food allergy, inhalation can also cause de novo sensitization [8], especially in occupational settings. Moreover, it is difficult to disentangle the distinct response from the entry route given that particles inhaled through the mouth will impact the back of the throat, and may be subsequently ingested [11].

Another factor that may contribute to a lower threshold for inhaled food allergens is that the allergenicity of milk proteins may be enhanced by the formation of lactose-protein complexes. Glycation of milk proteins occurs during heat treatment (Maillard reaction), leading to significant changes in the three-dimensional structure of these proteins. These conformational modifications might lead to large glycoprotein complex formation and potentially enhanced allergenicity, depending on certain factors. Intradermal skin test reactivity to β-lactoglobulin–lactose conjugates has been shown to increase by 10- to 100-fold compared with native β-lactoglobulin [17]. Nowak-Wegrzyn (2004) previously hypothesized that the random distribution of such large complexes may be responsible for differences in allergic reactions [16]. However, the effects of glycation and subsequent formation of aggregates do not always result in increased allergenicity, and this factor is largely dependent on the type or family of proteins. Caseins naturally tend to form ordered aggregates, which contributes to maintaining their IgE-binding capacity. However, when caseins form aggregates with other proteins, such as whey and wheat proteins, their IgE-binding capacity is increased or reduced, respectively [1].

In regard to the surface concentrations of lactose on the leaves of the plant, our results indicate that the amount of milk proteins after treatment inside and close to the treated area was of 10–100 μg/100 cm^2^. Milk proteins were still present on the surface of the leaves several days following spraying. Three days after treatment, they became very low, either due to leaf absorption or natural degradation. The simulation results indicate that surface contamination downwind of the vineyard could remain within the same order of magnitude of 100–200 m.

Taken together, these results demonstrate that exposure to airborne milk proteins concentrations, as well as those on the surface of the leaves, in and around treated agricultural areas is not negligible. This indicates a risk both for inhaled as well as ingested exposures. Previous reports have described children with severe milk allergy having acute allergic reactions after the ingestion of food products containing >10 parts per million of total milk protein. Moreover, foodborne allergies can be triggered by infinitesimal doses (from micro- to nanogram), irrespective of the route of exposure [18]. It is important to note that those who are allergic to food by ingestion may react to the same food by inhalation.

### Risk Characterization

The process of risk characterization considers the route of exposure, and assesses the probability of exposure, matched with the severity or the impact of that exposure. In this study, we can hypothesize that the likelihood of exposure will be low, but that the severity or health impact is potentially high.

A shown in Table 2, the risk by inhalation and ingestion can be considered as non-negligible. However, it is important to note that the time window of exposure opportunity can be very different for inhalation exposure and surface contact exposure for ingestion. The fact that exposure may occur outside the treated area or several hours or days after the actual treatment is a concern in tourist areas or near populated areas. Usual safety measures, which include prohibiting entry into the treatment area, similarly to any chemical treatments by helicopter or airplane, do not eliminate this risk. Considering that it only concerns a minority of the population, all-public measures, which would have to be taken several hundred meters from the treated area and several days after treatment, do not seem realistic. Targeted information messages to vulnerable populations, for example, through allergists, could be more effective and make skimmed milk treatments safer, as they are an interesting alternative to traditional, more polluting pesticides that are dangerous for humans and any other living thing (bees, birds, etc.).

## 5. Conclusions

Our results are the first to reveal that exposure to airborne milk proteins concentrations in and around treated agricultural areas is not negligible. As milk-allergy reactions can be triggered by milk protein concentrations in the nanogram range, these results provide important findings for vulnerable worker and community populations, particularly for children. This study presents the results of the first ever risk assessment of the use of milk as a PPP and provides novel information towards evidence-based occupational and public health policy. The usual safety measures used during treatment by plane or helicopter do not guarantee the protection of individuals with severe allergies, due to the spread of aerosols outside the treated area and their persistence in the air several hours after treatment. As this risk concerns a small proportion of the population, targeted information for people with severe allergies—concerning the risk of exposure to bovine proteins during the period of wine-growing treatments based on dairy products—should be considered.

## Figures and Tables

**Table 1:** AgDRIFT Parameters used in the downwind surface contamination simulation.

Parameter	Median Value	Upper/Lower Value	Description/Comment
Helicopter type	Eurocoper AS350 B2 (Ecuriel B3)	n.a.	The model parameters were adjusted to take into account the helicopter type.
Rotor radius	5.34 m	5.34 m	
Weight	1222 kg	1222 kg	The weight of Eurocoper AS350 B2
Rotor RPM	390 min^−1^	390 min^−1^	
Boom height (vertical)	3 m	2–4 m	This is the height of the helicopter while spraying.
Flight lines	10	10	Average number of rows of vines treated in one pass.
Sprayed product	Water	Water	This parameter is used to evaluate density and evaporation rate. Due to the small amounts of milk protein in the sprayed mixture, its effect on these parameters is negligible.
Wind speed	1 m/s (3.6 km/h)	1 m/s–3 m/s	A higher wind rate was considered for a wider dispersion of the product.
Wind direction	−90°	−90°	Conservative value.
Temperature	20 °C	20 °C	The actual temperature varies within the range of 16–20 °C. 20 °C is the most conservative value as it leads to a faster evaporation and smaller aerosols (remain longer in suspension).
Humidity	65%	65%	Conservative value (less humidity, higher evaporation rate).
Atmospheric stability	Spraying by day Slight solar isolation:	Spraying by day Slight solar isolation:	

**Table 2:** Risk characterization summary.

Entry Route	Exposure	Severity	Conclusion
Inhalation	– Average concentration of 0.62 µg/m^3^ – May–July (approximately 6 times per year) – Numerous public paths and a high likelihood of foot traffic – However, only a small prevalence of milk allergies in the population	Dose response: likely that allergic reactions are triggered even at doses in the nanogram range	Non-negligible risk by inhalation
Ingestion	– Average concentration of 107.5 µg/m^3^ – May–July (approximately 6 times per year) – Children exhibit hand-mouth behavior – Numerous public paths and a high likelihood of foot traffic – However, only a small prevalence of milk allergies in the population	Dose response: likely that allergic reactions are triggered even at doses in the nanogram range	Non-negligible risk by ingestion.

## Data Availability

The data from this study have all been presented as graphs or tables in the article. The raw data are available on request from the corresponding author.

## Supplementary Materials

The following are available online at https://www.mdpi.com/1660-4601/18/5/2400/s1, Figure S1: Picture of treated leaves, collected one day after treatment.
