# Supplementary figures and images for: Skimmed Milk Applied as a Phytopharmaceutical Product: A Risk for Allergic Populations?
Source: Int J Environ Res Public Health. 2021 Mar 1;18(5):2400. doi: 10.3390/ijerph18052400 (PMC7967751; doi:10.3390/ijerph18052400)

## Supplementary material

Figure 1S. Detailed view of the sampling area.

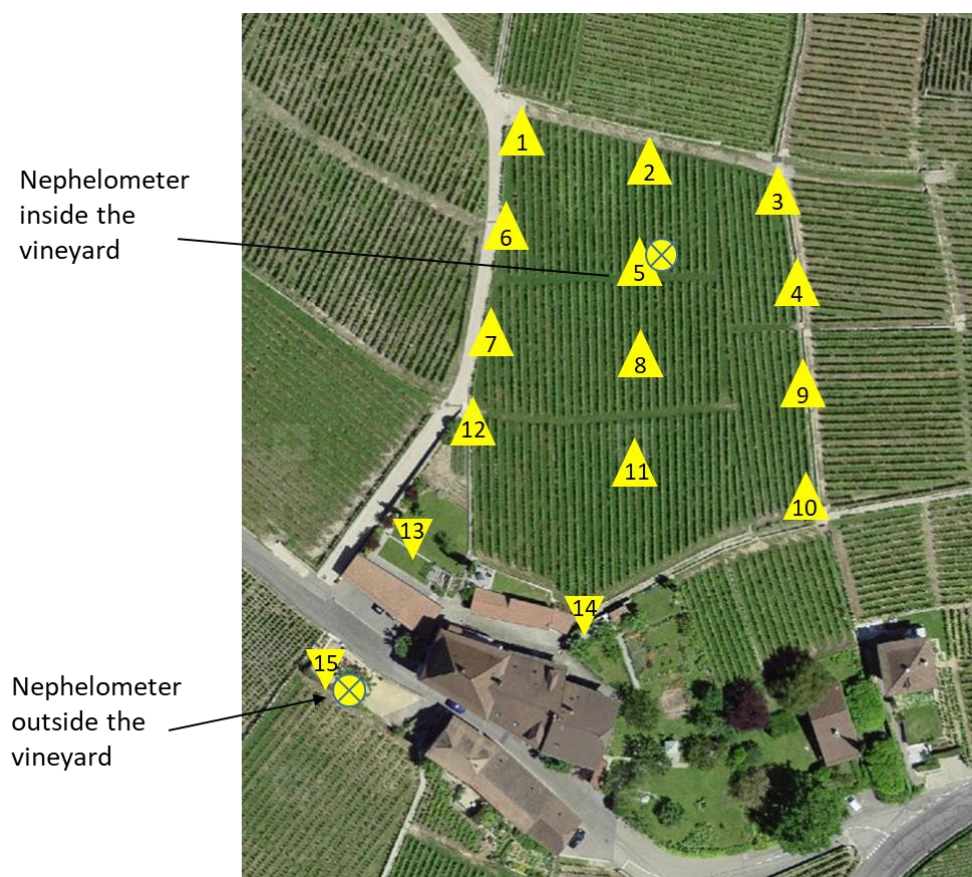

Supplement: Supplementary file 1 [file ijerph-18-02400-s001.pdf]
